# In Silico Screening of Multi-Domain Targeted Inhibitors for PTK6: A Strategy Integrating Drug Repurposing and Consensus Docking

**DOI:** 10.3390/ph17010060

**Published:** 2023-12-29

**Authors:** Yujing Zhou, Ming Wah Wong

**Affiliations:** Department of Chemistry, National University of Singapore, 3 Science Drive 3, Singapore 117543, Singapore; chmyuji@nus.edu.sg

**Keywords:** PTK6, drug repurposing, consensus docking, structure based virtual screening, in silico studies

## Abstract

Protein tyrosine kinase 6 (PTK6), also known as breast tumor kinase (BRK), serves as a non-receptor intracellular tyrosine kinase within the Src kinases family. Structurally resembling other Src kinases, PTK6 possesses an Src homology 3 (SH3) domain, an Src homology 2 (SH2) domain, and a tyrosine kinase domain (SH1). While considerable efforts have been dedicated to designing PTK6 inhibitors targeting the SH1 domain, which is responsible for kinase activity in various pathways, it has been observed that solely inhibiting the SH1 domain does not effectively suppress PTK6 activity. Subsequent investigations have revealed the involvement of SH2 and SH3 domains in intramolecular and substrate binding interactions, which are crucial for PTK6 function. Consequently, the identification of PTK6 inhibitors targeting not only the SH1 domain but also the SH2 and SH3 domains becomes imperative. Through an in silico structural-based virtual screening approach, incorporating drug repurposing and a consensus docking approach, we have successfully identified four potential ligands capable of concurrently inhibiting the tyrosine kinase domain and SH2/SH3 domains of PT6K simultaneously. This finding suggests potential pathways for therapeutic interventions in PTK6 inhibition.

## 1. Introduction

**Figure 1:**
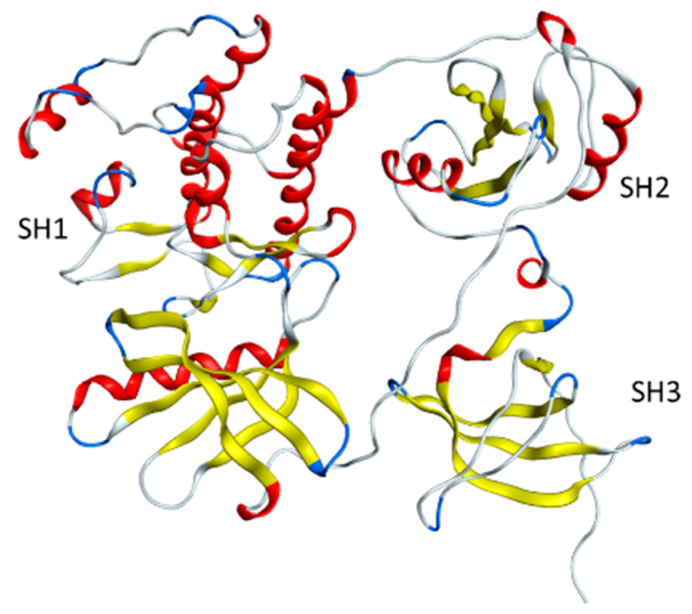
Structure of full PTK6 protein, encompassing an Src homology 3 (SH3) domain, an Src homology 2 (SH2) domain, and a tyrosine kinase domain (SH1).

Protein tyrosine kinase 6 (PTK6), also known as breast tumor kinase (BRK) [1,2,3], functions as an intracellular signal transducer in epithelial tissues [4]. BRK has been detected in some low-level breast tumors, and cells with overexpressed BRK become sensitive to epidermal growth factor, leading to a partially transformed phenotype [5]. As a member of the Src kinases family, PTK6 shares structural similarities with other Src kinases, encompassing an Src homology 3 (SH3) domain, an Src homology 2 (SH2) domain, and a tyrosine kinase domain (SH1) [6] (Figure 1). While considerable research effort has focused on designing inhibitors for the SH1 domain due to its kinase activity in various pathways [7,8,9], recent studies have indicated that *PTK6* functions in a kinase-independent manner and has complex, context-specific functions in some cancers [10,11]. Further investigations revealed that the SH2 domain binds to substrate phosphotyrosine motifs and enhanced protein–protein recognition and interactions [12,13]. Additionally, Trp44 in the SH3 domain and Pro177, Pro175, and Pro179 in the *N*-terminal half of the Linker region play important roles in maintaining an inactive conformation of the protein along with the phosphorylated Tyr447-SH2 interaction [13,14,15]. Both lines of evidence suggest that solely inhibiting the SH1 domain is not sufficient to suppress the activity of PTK6, and it is crucial to target SH2 and SH3 domains as well.

In this computational study, we utilized an integrated strategy of drug repurposing and consensus docking to search for potential inhibitors for PTK6. Drug discovery and development in a traditional way includes several stages for the successful discovery of a new drug. Drug repurposing, also known as drug repositioning, is a state-of-the-art strategy to reveal new therapeutic uses for identifying existing clinically used drugs with a substantial improvement of the development timeline. The advantages of the drug repurposing strategy are manifold. Firstly, as the formulation and preclinical safety for repurposed drugs have been tested and accomplished, this approach substantially reduces the time required for drug development. Secondly, there is a lower financial investment needed for drug development using the drug repurposing strategy. Preclinical and phase I and II costs are markedly reduced for a repurposed drug. Thirdly, and most importantly, the repurposed drug has been proved to be moderately safe in previous clinical trials, so the rate of failure due to safety concerns can be dramatically reduced. As a consequence, over the last few years, drug repurposing involves discovering new applications for existing drugs, and it has been shown to be a promising strategy for a more efficient and cost-effective pathway to discover innovative treatments [16,17,18].

Repurposing can be achieved by trial and error or a systematic approach. In this investigation, we employed a structure-based virtual screening strategy to introduce a more rational and systematic approach to drug repurposing. To this end, the 2016 FDA-approved drugs were used as a virtual library, screening against the three-dimensional structure of the PTK6 protein. Molecular docking, a process that involves the docking of ligands into the binding site of a particular receptor and using the binding pose and conformation to calculate binding affinities, was used to computationally “dock” ligands into the binding site of target protein. However, predicting the correct binding pose and calculating the accurate binding affinities pose significant challenges. Incorrectly predicted binding poses and binding affinities can significantly impact the success rate of drug repurposing predictions. Various docking software tools have different placement methods to predict docking poses/conformations as well as different scoring functions to calculate binding affinities. Therefore, we embraced a consensus docking approach, involving combining the results of multiple docking simulations. This approach aims to enhance the accuracy and reliability of predictions. This strategy is particularly relevant here given the current lack of known effective PTK6 inhibitors. Numerous studies highlight that the accuracy of scoring depends on the precision of the docking process, emphasizing the importance of considering the diverse docking placement methods and scoring functions employed by different docking methods rather than relying on a single scoring algorithm alone [19,20]. Hence, we adopted a consensus docking approach in our structure-based virtual study.

In our investigation, we implemented a drug repurposing strategy with the aim to identify potential leads capable of targeting multiple domains of the PTK6 protein, specifically SH1, SH2, and SH3. The methodology we employed involved a virtual screening process that hinged on the intricate details of the protein structure of PTK6, utilizing a consensus docking approach to improve the accuracy of prediction. Our in silico study successfully identified four potential ligands exhibiting inhibitory capabilities toward the SH1 domain and SH2/SH3 domains simultaneously, suggesting a promising therapeutic approach to inhibit the kinase activities of *PTK6*.

## 2. Results and Discussion

### 2.1. SH1 Domain Virtual Screening

**Figure 2:**
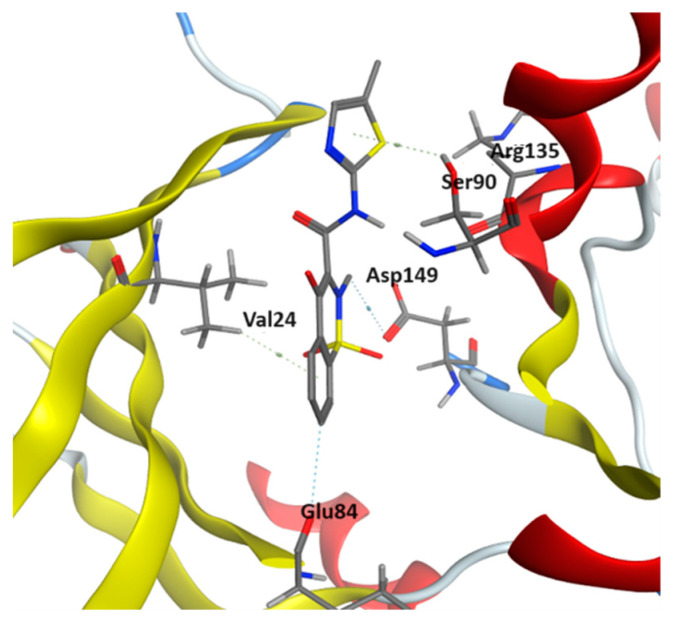
Structure of ligand Meloxicam docked to PTK6 kinase SH1 domain in MOE, showing CH–π interactions with Val24 and Ser90, and HB interactions with Glu84, Arg135 and Asp149.

A total of 100 top-ranked ligands from DRDOCK drug repurposing [21] were docked into the PTK6 SH1 domain [6] using Autodock Vina [22,23], DockingPie [24] and MOE [25]. Out of 100 ligands, 20 ligands with the best scores were selected from each docking software. By comparing the top 20 ligands from each set, nine consensus ligands were identified to be top-ranked for all sets, namely Regorafenib, Vx-661, Indacaterol, Vemurafenib, Camptothecin, 10-hydroxycamptothecin, Niraparib, Yohimbine, and Meloxicam, with docking scores ranging from −9.07 to −10.05 kcal/mol (Table 1). The PTK6 in crystal structure 6CZ3 bound to the ligand (3-fluoro-4-{[6-methyl-3-(1H-pyrazol-4-yl)imidazo [1,2-a]pyrazin-8-yl]amino}phenyl)(morpholin-4-yl)methanone with key residues Leu16, Met86 and Asp149. All nine ligands were identified to bind to the SH1 domain at the same site through CH–π and hydrogen bonding (HB) interactions. The original ligand in 6CZ3 binds to the PTK6 receptor at Leu16, Met86, Arg135 and Asp149. Our identified ligands bind to the PTK6 receptor with the same amino acid residues with additional interactions detected involving Ser18, Val24, Thr83, Glu84, Ser90 and Asn136. Figure 2 illustrates the binding of the ligand Meloxicam to the SH1 domain via CH–π interactions with residues Val24 and Ser90 as well as hydrogen bond (HB) interactions with residues Glu84, Arg135 and Asp149.

The highest ranked ligand Indacaterol binds to the PTK6 SH1 domain at amino acid residues Ser18, Leu16 and Thr83 via CH–π and hydrogen bonding interactions with a binding affinity of −10.05 kcal/mol. The second-ranked ligand 10-hydroxycamptothecin binds to the receptor at amino acid residues Val24, Arg135 and Asp149 via CH–π and hydrogen bonding interactions with a binding affinity of −10.03 kcal/mol. As for the third-ranked ligand Regorafenib, it binds to the PTK6 SH1 domain at Val24 and Met86 via similarly CH–π and hydrogen bonding interactions with −9.74 kcal/mol in binding affinity.

### 2.2. SH2 Domain Virtual Screening

For the SH2 domain [26], since the binding site is not well defined in the literature, web-based server CavityPlus [27] was utilized to detect the binding site. Based on cavity information from CavityPlus, residues 74–78 were selected as the target site for drug repurposing using DRDOCK. The top-ranked 100 ligands from DRDOCK were docked into the PTK6 SH2 domain using Autodock Vina, DockingPie and MOE. Out of 100 ligands, 20 ligands with the best score were selected for each docking software. By comparing the top 20 ligands, 13 consensus ligands were found to be top ranked for all sets, namely Leucovorin, Lifitegrast, Lumacaftor, 1370468-36-2, Zafirlukast, Fluralaner, Telmisartan, Nintedanib, Azilsartan Medoxomil, Daclatasvir, Aclacinomycin A, Epirubicin, and Doxorubicin, with docking scores ranging from −7.86 to −9.81 kcal/mol (Table 2).

**Figure 3:**
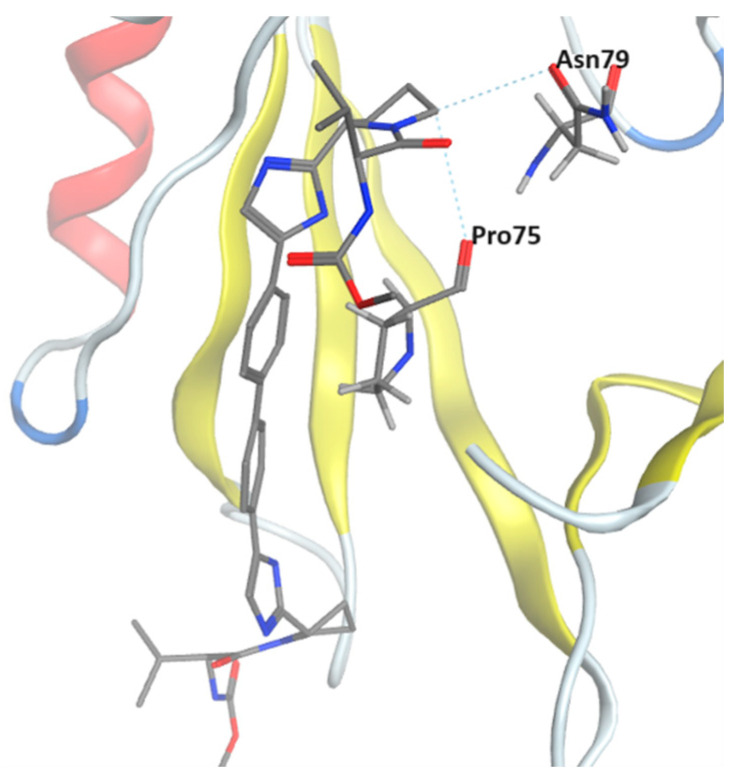
Structure of lead ligand Daclatasvir bound to PTK6 kinase SH2 domain in MOE, showing HB interactions with Pro75 and Asn79.

Among the 13 ligands, two ligands Fluralaner and Telmisartan bound to the PTK6 SH2 domain via hydrophobic interactions, while the rest of the ligands were identified to bind to the SH2 domain via CH–π and hydrogen bonding (HB) interactions. Key amino acid residues from the receptor—Pro03, Tyr40, Val50, Tyr53, Lys54, Arg5, Leu74, Pro75, and Asn79—were identified as contributing to the binding of ligands into the PTK6 SH2 domain. Figure 3 illustrated ligand Daclatasvir binding to the SH2 domain via HB interactions with residues Pro75 and Asn79.

The top-ranked ligand Lifitegrast binds to the PTK6 SH2 domain through CH–π and hydrogen bonding interactions at amino acid residues Tyr40, Leu74, Arg57 and Asp39, with a binding affinity of −9.81 kcal/mol. In contrast, the second-ranked ligand Lumacaftor, with a binding affinity of −9.58 kcal/mol, lacks the CH–π interaction, but it binds to the receptor at amino acid residues Pro3 and Val78 via hydrogen bonding interactions. The third-ranked ligand, Zafirlukast, binds to the PTK6 SH2 domain at Pro3, Glu2 and Gly7 via hydrogen bonding interactions with −9.74 kcal/mol in binding affinity. Similar to Lumacaftor, Zafirlukast also lacks the CH–π interaction.

### 2.3. SH3 Domain Virtual Screening

**Figure 4:**
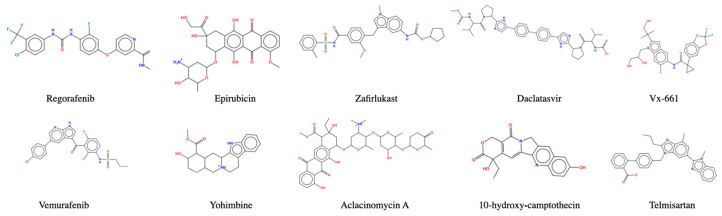
The top 10 ligands identified through consensus docking.

**Figure 5:**
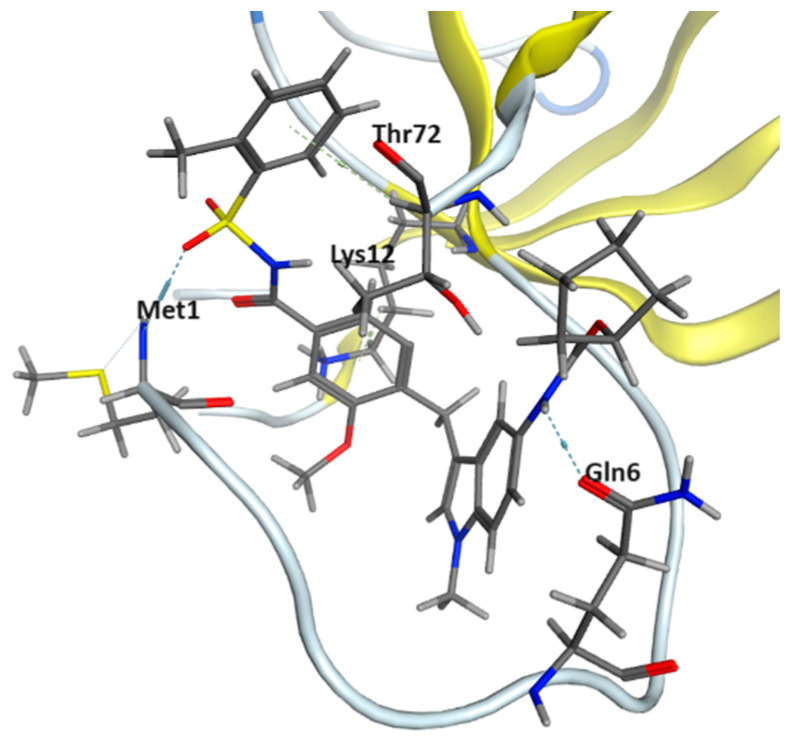
Ligand Zafirlukast bound to the PTK6 kinase SH3 domain in MOE, showing CH–π interactions with Lys12 and Thr72 as well as HB interactions with Met01 and Gln06.

Concerning the SH3 domain [28], given the lack of well-defined binding sites in the literature, web-based server CavityPlus was utilized to identify potential binding sites. However, all three detected binding cavities exhibited weak drug score and druggability. Since there are no common ligands that bind with both the SH1 and SH2 domains, the 100 top-ranked ligands with the SH3 domain from DRDOCK were compared with consensus ligands from the SH1 and SH2 domains, respectively. Five ligands were identified to bind to both the SH1 and SH3 domains, namely Regorafenib, Vemurafenib, Vx-661, 10-hydroxycamptothecin, and Yohimbine (Figure 4). Additionally, five ligands were found to bind to both the SH2 and SH3 domains, namely Aclacinomycin A, Epirubicin, Zafirlukast, Telmisartan, and Daclatasvir (Figure 4). The docking scores of these 10 ligands with the SH3 domain calculated using MOE are listed in Table 3. In Figure 5, the illustration depicts the binding of the ligand Zafirlukast to the PTK6 kinase SH3 domain via CH–π interactions with Lys12 and Thr72, and HB interactions with Met01 and Gln06.

**Figure 6:**
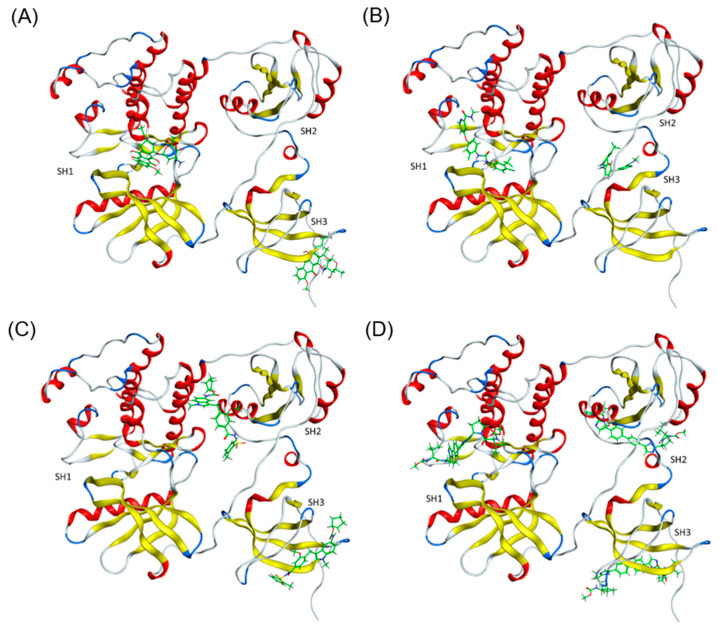
Lead ligands bound to multiple domains. (**A**) Epirubicin bound to SH1 and SH3 domains. (**B**) Regoragenib bound to SH1 and SH3 domains. (**C**) Zafirlukast bound to SH2 and SH3 domains (**D**) Declatasvir bound to SH1, SH2 and SH3 domains.

The highest-ranked ligand Aclacinomycin A has a binding affinity of −8.62 kcal/mol. However, it binds to only two amino acid residues at the SH3 domain. The second-ranked ligand Daclatasvir, with a binding affinity of −7.98 kcal/mol, binds to the PTK6 SH3 domain with the most amino acid residues compared to other ligands. Ser03, Pro11, Lys12, Arg70 and Thr72 collectively contribute to the overall binding score. Epirubicin, Zafirlukast and Telmisartan, with binding affinities of −7.04 kcal/mol, −7.02 kcal/mol and T he location of Figure 6 is fine −7.27 kcal/mol, respectively, bind to the PTK6 SH3 domain with amino acid residues Met01, Gln06, His08, Lys12 and Thr72.

### 2.4. Full Protein Docking

Finally, molecular docking with the full PTK6 protein using MOE was performed for the top 10 lead ligands (Figure 4) to validate their binding preferences across multiple domains. The top-ranked 15 poses were analyzed for each ligand. The binding scores and domains of the 10 ligands are listed in Table 4. Epirubicin (Figure 6A) and Regorafenib (Figure 6B) demonstrated binding to the SH1 and SH3 domains, while Zafirlukast (Figure 6C) exhibited binding to the SH2 and SH3 domains. Intriguingly, Declatasvir (Figure 6D) displayed binding to all three domains with similar binding scores. These four ligands capable of binding to multiple domains exhibited the best binding scores. On the other hand, VX-611, Vemurafenib, and Yohimbine showed binding at the center of the protein with moderate binding scores only, indicating a lesser preference toward the full protein. Aclacinomycin A and 10-hydroxycamptothecin, despite high binding scores for full protein binding, tend to bind solely to the SH1 domain only, which is contrary to our goal to target multiple domains. Telmisartan bound exclusively to SH3, yielding a less favorable binding score.

In summary, four ligands exhibited a preference for binding to multiple domains with favorable scores (Table 4). Specifically, Epirubicin and Regorafenib bind to the SH1 and SH3 domains with binding scores of −6.48 kcal/mol and –7.33 kcal/mol, respectively. Zafirlukast shows affinity to the SH2 and SH3 domains, with a binding score −6.42 kcal/mol, while Declatasvir binds to all three domains with a binding score of −6.57 kcal/mol. These results indicate a successful prediction of multi-domain targeted inhibitors for PTK6, employing an integrated approach that combines drug repurposing and structure-based virtual screening through consensus docking methods.

Regorafenib functions as an orally administered inhibitor of multiple kinases, demonstrating anti-angiogenic activity through its dual-targeted VEGFR2-TIE2 tyrosine kinase inhibition. This drug is employed in the treatment of metastatic colorectal cancer, advanced gastrointestinal stromal tumors, and hepatocellular carcinoma [29]. Epirubicin, an anthracycline topoisomerase II inhibitor, exerts its antitumor effects by disrupting the synthesis and function of DNA. It serves as an adjuvant in treating axillary node metastases for patients who have undergone surgical resection of primary breast cancer [30]. Zafirlukast, classified as an oral leukotriene receptor antagonist (LTRA), blocks the action of cysteinyl leukotrienes on the CysLT1 receptors. This drug is utilized for the maintenance treatment of asthma [31]. Daclatasvir, a direct-acting antiviral agent against Hepatitis C Virus (HCV), targets both cis- and trans-acting functions of NS5A. By modulating the NS5A phosphorylation status, it disrupts the function of new HCV replication complexes. Daclatasvir is prescribed for the treatment of chronic HCV genotype 1 and 3 infections [32].

As a member of the Src kinase family, the non-receptor intracellular tyrosine kinase Protein tyrosine kinase 6 (PTK6) comprises an Src homology 3 (SH3) domain, an Src homology 2 (SH2) domain, and a tyrosine kinase domain (SH1). It is observed that by solely blocking PTK6 phosphorylation and make it catalytically inactive, leaving the SH2 and SH3 domains free to interact with other substrates, breast cancer cells are still able to proliferate. While considerable efforts have been dedicated to designing PTK6 inhibitors targeting the SH1 domain, which is responsible for kinase activity in various pathways, it has been observed that solely inhibiting the SH1 domain does not effectively suppress PTK6 activity. Further investigations have revealed the involvement of SH2 and SH3 domains in intramolecular and substrate binding interactions, which play an essential role in the functionality of PTK6. Free SH2 and SH3 domains may still potentially promote cancer cells’ proliferation. Consequently, it becomes imperative to identify PTK6 inhibitors that not only target the SH1 domain but also effectively interact with the SH2 and SH3 domains to comprehensively inhibit PTK6 activity. This holistic approach is crucial for developing more effective strategies to curb the proliferative potential of cancer cells associated with aberrant PTK6 function. The four ligands identified here show promise as potential candidates for such multi-domain targeted inhibitors.

## 3. Materials and Methods

### 3.1. Potential Binding Sites of SH2 and SH3 Domains Using CavityPlus

Unlike the tyrosine kinase domain SH1, the PTK6 SH2 and SH3 domains lack information regarding ligand binding and cavities. Before engaging in ligand docking, we used a web-based server named CavityPlus [27] to identify potential binding cavities for SH2 and SH3 domains. PDB structures of SH2 (1RJA) [26] and SH3 (2KGT) [28] were uploaded to the CavityPlus web server: chain A was selected, the ligand-free mode was implicated, and other parameters served as the default. In the case of the SH2 domain, four cavities were detected and ranked using drug score and druggability. The top-ranked cavity #1 was chosen for subsequent docking steps. Similarly, for the SH3 domain, three cavities were detected with ranked based on drug score and druggability with the top-ranked cavity #1 chosen for the next stage of docking.

### 3.2. Docking with FDA-Approved Drugs

DRDOCK is a web-based server designed for the virtual screening of 2016 FDA-approved drugs on the user-submitted protein target [21]. It is worth noting that the performance assessment of DRDOCK revealed that the true binders are within the top-ranked 100 examined drugs [21]. The 2016 FDA-approved drugs were sourced and consolidated from the MedChemExpress (MCE) FDA-Approved Drug Library (Cat. No.: HY-L022) and Enzo Life Sciences, Inc. (Farmingdale, NY, USA) Screen-Well^®^ FDA Approved Drug Library (Version 1.5). There are a total of 2016 small molecule drugs encompassed within this library. These small molecule drugs were represented with structures created using BIOVIA Discovery Studio [33], considering the protonation states of ionizable groups at pH = 7. The input PDBQT files for drug docking were generated using AutoDock Tools [34]. In the case of SH1, PDB file 6CZ3 [10] was submitted with chain A selected for docking. Residues 85–91 were selected as the target site. For SH2, PDB file 1RJA was submitted with chain A selected for docking. Residues 74–78, which were detected from CavityPlus, were selected as the target site. Similarly, for SH3, PDB file 2KGT was submitted, with chain A selected for docking, and residues 36–40, which were detected from CavityPlus, were selected as the target site. Docking results for each domain were ranked with novel drug ranking method log-odds (LOD) scores combining feature distributions of true binders and decoys, including terms of docking affinity, contact number, distance to target site, cluster size and number of clusters. The top 100 ranked drugs were subsequently chosen for further consensus docking analysis.

### 3.3. Consensus Docking

The top-ranked 100 ligands from DRDOCK for SH1 and SH2 domains were selected for consensus docking using three different docking methods, namely Autodock Vina [22,23], DockingPie [24] and Molecular Operating Environment (MOE) [25]. For Autodock Vina, Autodock Tools 1.5.4 [34] were utilized for ligands and receptor preparation. Water molecules and other heteroatoms were removed, protonated hydrogen atoms and Gasteiger charges were assigned to atoms, and the grid box size was set to be 60 Å. PDBQT files of ligand structures were generated using OpenBabel 2.3.0 [35]. For Autodock Vina and DockingPie, the same prepared files were used for five runs of repeated docking with 10 poses each. For docking with MOE, a library of the top-ranked 100 ligands from DRDOCK were generated. Docking with the library with the Triangle Matcher placement method and rescoring was conducted with GBVI/WSA dG [36,37] scoring function to predict poses and scores. Docking results for each method were plotted, and the top 20 ligands from each set were compared. After the comparison, 10 consensus ligands were identified for the SH1 domain and 13 were identified for the for SH2 domain. In the case of the SH3 domain, the top 100 ligands from DRDOCK were used to compare with the ligands set from the SH1/SH2 domains, and the 10 lead ligands were identified that bind multiple domains.

### 3.4. Full Docking with MOE

Finally, the 10 lead ligands identified from consensus docking were docked into the complete PTK6 protein to check their binding conformations. Given the absence of an established crystallized structure for the entire PTK6 protein, we sourced the structure from the Alphafold Protein Structure Database, specifically from UniProt Q13882. Triangle matcher was selected as the placement method and the GBVI/WSA dG rescoring function was chosen to predict the binding score. The top 15 poses for each ligand were checked for binding-domain preference. Ligand–receptor interaction analysis was conducted using the MOE user–graphical interface.

## 4. Conclusions

A collection of 2016 FDA-approved drugs were screened virtually through consensus docking to identify potential lead ligands. This strategy capitalizes on the wealth of existing pharmacokinetics, pharmacodynamics, and safety data from human trials for FDA-approved drugs, streamlining the drug discovery process, saving time and reducing complexity. The top-ranking ligands from the structure-based virtual screening were further refined using a consensus docking approach that incorporated Autodock Vina, DockingPie, and MOE docking methods. The final selection was based on ranks obtained from individual docking programs. This integrated approach led us to identify 10 repurposed FDA-approved drugs which have promising binding affinity toward the PTK6 protein. After thorough full protein docking validation, four drugs, namely Epirubicin, Regorafenib, Zafirlukast, and Declatasvir, were identified for their remarkable ability to simultaneously bind to multiple domains of the PTK6 protein. We believe this discovery opens up potential new avenues for therapeutic interventions in PTK6 inhibition [38]. These findings not only underscore the potential efficacy of repurposing existing drugs for novel therapeutic applications but also contribute to the advancement of targeted interventions for PTK6-related conditions. The identification of multiple ligands capable of concurrently targeting distinct domains of PTK6 not only paves the way for further experimental validation but also holds promise for the development of more effective treatments specifically targeting this kinase.

## Figures and Tables

**Table 1:** Consensus ligands bind to SH1 domain, interactions with receptor residues and corresponding docking scores (kcal/mol).

Ligands	Interaction	Receptor Residues	Autodock Vina	DockingPie	MOE
Regorafenib	CH–π	Val24	−9.74	−8.81	−7.85
	HB	Met86			
Vx-661	CH–π	Ser90	−9.39	−9.33	−7.93
	HB	Ser18, Thr83, Ser90			
Indacaterol	CH–π	Ser18	−10.05	−9.92	−7.53
	HB	Leu16, Thr83			
Vemurafenib	CH–π	Val24	−9.07	−9.46	−7.39
	HB	Ser90			
Camptothecin	CH–π	Val24	−9.23	−9.31	−6.95
	HB	Asp149			
10-hydroxy-camptothecin	CH–π	Val24	−10.03	−9.51	−7.16
	HB	Arg135, Asp149			
Niraparib	CH–π	Val24	−9.62	−9.13	−7.21
	HB	Glu84, Asn136			
Yohimbine	CH–π	Leu16	−9.53	−9.17	−7.23
	HB	Asn136, Asp149			
Meloxicam	CH–π	Val24, Ser90	−9.59	−8.76	−7.11
	HB	Glu84, Arg135, Asp149			

**Table 2:** Consensus ligands bind to SH2 domain, detailing their interactions with receptor residues and corresponding docking scores (kcal/mol).

Ligands	Interaction	Receptor Residues	Autodock Vina	DockingPie	MOE
Leucovorin	CH–π	Pro75	−8.61	−7.16	−6.38
	HB	Tyr40, Arg57, Asn79			
Lifitegrast	CH–π	Tyr40	−9.81	−8.63	−6.53
	HB	Leu74, Arg57, Asp39			
Lumacaftor	HB	Pro3, Val78	−9.58	−8.87	−5.56
1370468-36-2	CH–π	Pro75	−8.96	−9.15	−7.38
	HB	Leu74			
Zafirlukast	HB	Pro3, Glu2, Gly7	−9.07	−7.97	−6.51
Fluralaner	Hyd Int ^1^		−9.01	−7.86	−6.59
Telmisartan	Hyd Int ^1^		−8.89	−8.79	−6.28
Nintedanib	CH–π	Phe5	−8.41	−8.21	−6.82
	HB	Pro75			
Azilsartan Medoxomil	HB	Ser73	−8.54	−7.24	−6.37
Daclatasvir	HB	Pro75, Asn79	−8.97	−8.03	−7.01
Aclacinomycin A	HB	Val50, Tyr53, Lys54	−7.89	−6.94	−6.54
Epirubicin	HB	Lys54	−7.86	−7.03	−5.95
Epirubicin	HB	Lys54	−7.86	−7.03	−5.95
Doxorubicin	CH–π	Tyr53	−8.09	−6.85	5.46
	HB	Lys54, Ser87			

^1^ hydrophobic interaction.

**Table 3:** Ligands bind to the SH3 domain, detailing their interactions with receptor residues and corresponding docking scores (kcal/mol).

Ligands	Receptor Resides	MOE Score
Regorafenib	Glu69	−6.67
Vemurafenib	Met01, Lys12	−6.66
Vx-661	Lys12	−7.45
10-hydroxy-camptothecin	no interactions detected	−5.95
Yohimbine	Trp45	−5.96
Aclacinomycin A	Met01, Lys12	−8.62
Epirubicin	Met01, His08, Lys12	−7.04
Zafirlukast	Met01, Gln06, Lys12, Thr72	−7.08
Telmisartan	Met01, Lys12	−7.27
Daclatasvir	Ser03, Pro11, Lys12, Arg70, Thr72	−7.98

**Table 4:** MOE binding scores (kcal/mol) of lead ligands bind to multiple domains of PTK6.

Ligands	SH1 Domain	SH2 Domain	SH3 Domain
Regorafenib	−7.33	NA	−5.87
Epirubicin	−6.48	NA	−5.96
Zafirlukast	NA	−6.42	5.91
Daclatasvir	−6.39	−6.41	−6.57
Vx-661		−6.11 ^1^	
Vemurafenib		−5.98 ^1^	
Yohimbine		−5.15 ^1^	
Aclacinomycin A	−6.84	NA	NA
10-hydroxy-camptothecin	−6.51	NA	NA
Telmisartan	NA	NA	−6.05

^1^ Bind at the center of protein.

## Data Availability

Data are contained within the article.
